# Characterization of Volcanic Ash Influence on the Nutritional Quality and Biological Traits in Potato Crops of the Cotopaxi Region

**DOI:** 10.3390/toxics13060453

**Published:** 2025-05-29

**Authors:** Raluca A. Mihai, Ramiro Fernando Vivanco Gonzaga, Nathaly Raquel Romero Balladares, Rodica D. Catana

**Affiliations:** 1 CIAM, Department of Life Science and Agriculture, Universidad de Las Fuerzas Armadas-ESPE, Av. General Ruminahui s/n y, Sangolqui 171103, Ecuador; 2 Department of Life Science and Agriculture, Universidad de Las Fuerzas Armadas-ESPE, Av. General Ruminahui s/n y, Sangolqui 171103, Ecuador; rfvivanco2@espe.edu.ec (R.F.V.G.); nrromero@espe.edu.ec (N.R.R.B.); 3 Institute of Biology Bucharest of Romanian Academy, 296 Splaiul Independentei, 060031 Bucharest, Romania; rodica.catana@ibiol.ro

**Keywords:** antioxidant activity, flavonoids, heavy metals, inductively coupled plasma (ICP), phenolic compounds, *Solanum tuberosum* L., volcanic ash

## Abstract

This study investigates the impact of volcanic ash from Cotopaxi Volcano on the nutritional quality and biological traits of potato tubers (*Solanum tuberosum* L.) cultivated in the Cotopaxi region. Methods: Samples collected from exposed and unexposed areas were used to characterize the volcanic ash influence on the metabolic aspects of the potato crop. The colorimetric method; DPPH, ABTS, and FRAP assays; and ICP-OES were used to better understand potatoes’ reaction to the stress. Results: Antioxidant activity was significantly higher (4.80 ± 2.38 µmol Trolox g^−1^ DW-DPPH assay; 11.05 ± 2.57 µmol Trolox g⁻^1^ DW-ABTS assay; and 11.96 ± 4.57 µmol Fe^2^⁺ g⁻^1^ DW-FRAP assay) in ash-exposed samples, suggesting enhanced stress responses. The bioactive compounds studied followed a comparable trend, with high content in the exposed tubers. Also, significant changes in elemental composition were registered: Potassium levels decreased in unexposed samples, while magnesium and iron levels increased. Metallic elements (zinc; lithium; boron; manganese; barium; lead; nickel; chromium; indium) were in concentrations <0.01 mg/kg. Conclusion: These findings demonstrate that volcanic ash alters the metabolic and antioxidant profiles of potato tubers, enhancing nutraceutical properties while posing food safety risks due to heavy metals. This dual impact highlights the challenges and opportunities for agriculture in volcanic regions like Cotopaxi.

## 1. Introduction

Potatoes are among the most widely grown crops worldwide, due to their high yield potential and ability to grow in diverse climatic conditions [1]; they are recognized as a vital resource in nutrition [2] and agricultural versatility.

For the Andean region, potato tubers are one of the most important crops, where diverse environmental factors contribute to their development and quality. The Andean region is a treasure trove of potato genetic diversity, which is crucial for ensuring the resilience of potato crops to pests, diseases, and climate change [3].

Comprehending the interplay between volcanic ash and plant development is important to assess the risks to agriculture and public health in volcanic regions. The Cotopaxi Volcano, one of the most active volcanoes in the world, emits ash characterized by a unique composition of minerals and heavy metals that significantly influences the surrounding agricultural lands, with both beneficial and detrimental aspects [4]. According to Smith and Lee, 2020 [5], volcanic ash contains silica, aluminum, iron, calcium, and magnesium, with reported increases of 20–30% in phenolic content in crops exposed to volcanic soils [6]. We hypothesize that volcanic ash from Cotopaxi increases the antioxidant profiles of potatoes, improving their nutraceutical quality, although it could increase food safety risks due to heavy metal accumulation. These elements can alter soil properties, potentially affecting the morphological and chemical characteristics of crops grown in ash-affected regions. Heavy metals pose particular concerns, as they may be absorbed by potato tubers, thus entering the food chain and potentially compromising food safety [7].

Few studies are available on the influence of volcanic ash on the nutritional quality of potatoes. Ligot et al., 2025 [8], point out that current models for predicting the impact of ash on crops use only ash thickness (or mass load) as a variable, without taking into account other factors (plant traits; growth stage; metabolic profiles; etc.); their results have been used to develop new vulnerability functions for estimating yield loss in potatoes exposed to an ash fall event. Other studies have shown that crops cultivated in soil improved with volcanic ash, showing an increased yield, crude protein, starch content in potato tubers, and also better resistance to potato diseases [9,10]. In this context, our study examines the impact of ash originating from the Cotopaxi Volcano on potatoes cultivated under two different conditions: exposed and unexposed to volcanic ash. By analyzing the antioxidant content and examining the overall impact of volcanic ash on potato tubers, this research provides crucial insights into how volcanic activity affects local agriculture. It highlights not only the challenges faced by farmers in volcanic regions but also the potential opportunities to enhance the functional and health-promoting qualities of staple crops like potatoes. However, it also addresses the risk of compromised food security at a national level due to the presence of heavy metals in volcanic ash. These findings are particularly significant for the Cotopaxi region, where volcanic activity continues to influence both the environment and the livelihoods of its inhabitants.

This study emphasizes the need for further research into optimizing agricultural practices in volcanic regions, balancing the advantages of improved crop quality with potential risks to food security. By understanding the complex interactions between soil properties and plant metabolism, agricultural systems in volcanic areas can be better managed to ensure both productivity and crop security.

## 2. Materials and Methods

Potato tubers (*Solanum tuberosum* L.) were collected from two distinct areas in the Cotopaxi region, Ecuador: areas exposed to volcanic ash (CC1 and CC2) and unexposed areas (CS1 and CS2). The areas are separated by 20 km and are approximately 60 km from the Cotopaxi volcano. The exposed samples (CC1 and CC2) came from agricultural fields in the parish of Guasaganda, affected by emissions from Cotopaxi, while the unexposed samples (CS1 and CS2) were collected from fields in the parish of Mulaló, without direct influence of recent volcanic ash. The collection was carried out during the harvest season, selecting mature tubers from plants cultivated under traditional local agricultural practices.

Sampling areas were randomly selected within representative agricultural fields with recent ash exposure (post 2015). Exposed soils had an average pH of 6.2, 3.5% organic matter, and high mineral load (e.g., Fe: 120 mg/kg), versus pH 6.8 and 2.8% in unexposed soils. ICP-OES analysis had detection limits of 0.01 mg/kg and recoveries of 95–105% for multielement standards.

Bioactive compound extraction was conducted according to the procedure outlined by Claros, 2021 [11], with modifications adapted for *S. tuberosum.* Fresh and mature potato tubers were processed by grinding them in a mortar to obtain a homogeneous paste. Subsequently, 1 g of the paste was precisely weighed using an analytical balance and subjected to maceration with 96% ethanol (10 mL) in 15 mL Falcon tubes. The mixture was manually stirred using a glass rod and then refrigerated at 5 °C for 72 h to optimize the extraction. The absorbance was quantified using a UV-Vis spectrophotometer (Thermo Fisher Scientific, Waltham, MA, USA). All biochemical experiments (extractions and determinations) were carried out in triplicate.

The phenolic content in samples collected from both sites with and without volcanic ash *S. tuberosum* tubers was quantified using the Folin–Ciocalteu colorimetric method, following the protocol described by López-Froilán et al., 2018 [12]. To determine the total phenolic compounds, a volume of 1.0 mL diluted potato extract (1:10) or standard solution was mixed with Folin–Ciocalteu reagent (same volume) and a small volume of distilled water. In total, 4 mL of Na_2_CO_3_ (100 mg/L) was added after 4 min, and the volume was adjusted to 25 mL using distilled water. A spectrophotometer was used to detect absorbance at 750 nm after the solution was incubated for 90 min at room temperature in the dark. Ethanol was used as a blank, with results given in mg GAE/L, using a calibration curve based on gallic acid (0–250 mg GAE/L).

For flavonoid quantification, Pekal et al.’s [13] method was used, which consists of mixing crude potato extract (1 mL), solvent (1.5 mL), CH_3_COONa 1 M (100 µL), AlCl_3_ 10% *v*/*v* (100 µL), and distilled water (2.3 mL). The mixture was left to rest for 40 min at room temperature before measuring absorbance at 435 nm.

The antioxidant capacity evaluation through FRAP assays was based on the methodology of Agudo, 2010 [14]. As amount of 593 nm was used to measure the absorbance. FRAP reagent was prepared by mixing acetate buffer 300 mM, pH 3.6 (100 mL), FeCl_3_ 20 mM (10 mL), and distilled water (12 mL). Each reaction consisted of FRAP reagent (300 µL), potato extract (100 µL), and distilled water (300 µL), incubated for 4 min, in the dark, at room temperature. Ethanol was used as a blank, with a standard curve y = 0.5981x − 0.0082 with R^2^ = 0.9989.

The Ramírez, 2023 [15] protocol was used for DPPH assay, which consists of mixing DPPH reagent (2.9 mL) with sample (0.1 mL) and maintaining the mixture for 30 min in the dark. As amount of 517 nm was used to measure the absorbance. Ethanol was used as a blank, with a standard curve y = 18.073x + 1.2252 with R^2^ = 0.9868.

For the ABTS assay, the methodology described by Mendoza, 2018 [16], was followed, generating the ABTS•+ radical, and adjusted at an absorbance of 0.76 ± 0.1 at 754 nm. A mixture of ABTS•+ solution (980 µL) and extract (20 µL) was incubated for 7 min at room temperature, and absorbance was measured at 754 nm. A positive control represented by Trolox was used, with a standard curve y = 34.102x + 9.2946 with R^2^ = 0.9612.

For the Inductively Coupled Plasma (ICP) analysis, dry samples of *S. tuberosum* tubers collected from soil control (SC) and post-Cotopaxi eruption conditions (PCCs) were prepared by weighing 0.5 g of finely ground plant material and transferring it into high-resistance Teflon tubes. A reagent mixture composed of nitric acid (HNO_3_), hydrogen peroxide (H_2_O_2_), and ultrapure water was added to each tube. The samples underwent a microwave-assisted digestion process following a standardized program for plant materials, as described in [16,17]. After digestion, the resulting solutions were filtered and diluted with ultrapure water in volumetric flasks, preparing them for trace element analysis. A 10 mL volume was used for the following analysis. Trace element quantification was conducted using an ICP-OES Thermo Fisher Scientific 7400 Duo (Waltham, MA, USA) (Inductively Coupled Plasma Optical Emission Spectrometer). A multielement ICP Mix 33 standard (0.01–7.5 mg/L) and 5 ppm yttrium (as an internal standard) were used for calibration and to account for potential instrumental variations and enhance analytical precision [18,19].

With a significance level of *p* < 0.05, two-factor ANOVA was used to evaluate significant differences between experimental groups in the statistical analysis, which was carried out using RStudio software version 4.3.2. Every experiment was carried out in triplicate, and the mean ± standard deviation (SD) was used to express the results. Pearson’s correlation coefficient was used to assess the relationship between antioxidant capacity and secondary metabolite concentration. Bar graphs with error bars used to visualize data were generated using the ggplot2 package, ensuring an accurate and clear representation of the observed variations. Additionally, a heatmap was constructed to illustrate the distribution of trace elements obtained from the ICP-OES analysis, facilitating comparative evaluation across samples affected and unaffected by volcanic ash.

## 3. Results

**Figure 1:**
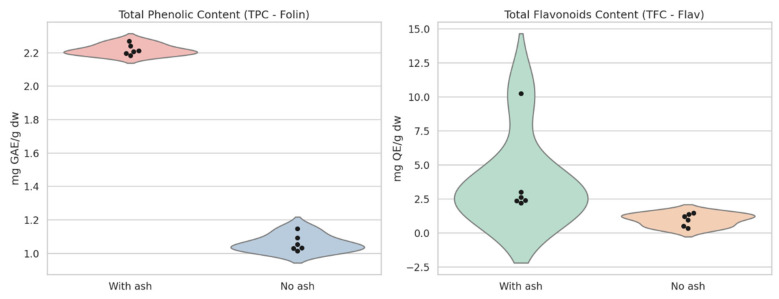
Bioactive compounds analyzed in the potato samples cultivated from exposed and unexposed areas in the Cotopaxi region. The black dots represent individual measurements, providing insight into the variability within each group.

The TPC values in samples collected from the ash-exposed place (CC1) varied between 2.206 and 2.270 mg GAE/g DW. Similarly, CC2 samples displayed TPC values varying between 2.183 and 2.211 mg GAE/g DW. In contrast, the samples collected from the non-ash-exposed place (CS1) exhibited lower TPC values with an average of 1.070 ± 0.054 mg GAE/g DW. The CS2 samples showed a similar trend, with TPC values with a mean of 1.053 ± 0.039 mg GAE/g DW (Figure 1).

TFC values followed a distinct pattern among the samples. CC1 samples exhibited TFC values ranging from 2.229 to 2.635 mg QE/g DW, with an average of 2.428 ± 0.168 mg QE/g DW. In CC2, a notable variation was observed, with TFC values ranging from 2.387 to 10.248 mg QE/g DW, averaging 5.884 ± 4.011 mg QE/g DW, suggesting significant metabolic fluctuations potentially influenced by volcanic ash exposure. CS1 samples presented TFC values between 0.980 and 1.464 mg QE/g DW (1.272 ± 0.200 mg QE/g DW average), while CS2 exhibited the lowest TFC values (0.372–1.216 mg QE/g DW), with an average of 0.698 ± 0.436 mg QE/g DW. The variability in TFC of CC2 (2.387 to 10.248 mg QE/g DW) could reflect soil heterogeneity or differential metabolic responses, with no statistical outliers identified (Grubbs test, *p* > 0.05). The negative DPPH value in CS1 (−0.12 µmol Trolox g^−1^ DW) indicates possible assay interference, adjusted to zero for further analysis (Figure 1).

**Figure 2:**
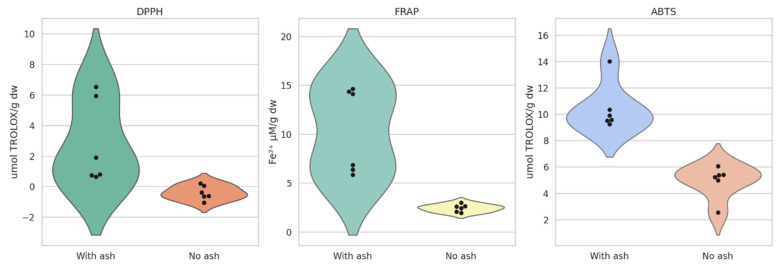
Antioxidant activity in potato samples cultivated with and without volcanic ash in the Cotopaxi region. Legend: DPPH—α-diphenyl-α-picrylhydrazyl free radical scavenging; FRAP—ferric-reducing antioxidant power; ABTS—free radical scavenging activity. The black dots represent individual measurements, providing insight into the variability within each group.

The antioxidant capacity of potato samples cultivated under volcanic ash influence in the Cotopaxi region, obtained using three different assays, showed that samples collected from ash-exposed areas (CC1 and CC2) exhibited significantly higher antioxidant activity across all assays compared to samples collected from non-ash places (CS1 and CS2). In the DPPH assay, CC2 recorded the highest activity (4.80 ± 2.38 µmol Trolox g⁻^1^ DW), while CS1 displayed negative values. Similarly, ABTS results confirm higher antioxidant potential in CC2 (11.05 ± 2.57 µmol Trolox g⁻^1^ DW), whereas CS1 and CS2 exhibited significantly lower activity. The FRAP assay, which measures reducing power, also showed a clear difference between samples. CC1 had the highest reducing capacity (11.96 ± 4.57 µmol Fe^2^⁺ g⁻^1^ DW), followed closely by CC2, while CS1 and CS2 presented the lowest values (Figure 2).

**Figure 3:**
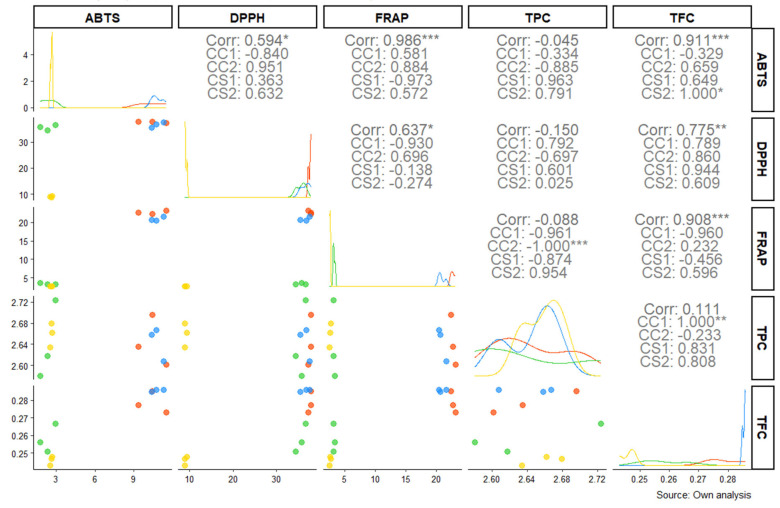
Correlation matrix between bioactive compounds and antioxidant capacity in potato cultivated in Cotopaxi region. The plot includes density distributions along the diagonal and scatterplots with regression lines for each pairwise comparison. Legend: CC1, CC2—samples collected from volcanic ash-exposed place; CS1, CS2—samples collected from non-ash-exposed place; TPC—total phenolic content; TFC—total flavonoid content; DPPH—α-diphenyl-α-picrylhydrazyl free radical scavenging; FRAP—ferric-reducing antioxidant power; ABTS—free radical scavenging activity; r—correlation coefficient; * *p* < 0.05; ** *p* < 0.01; *** *p* < 0.001. Different colors indicate data for each sample; lines represent the fitted correlation trend for the respective assays.

The correlation analysis between the antioxidant properties of potato samples cultivated in the Cotopaxi region (Figure 3) revealed distinct patterns among the different antioxidant assays (DPPH, FRAP, ABTS) and bioactive compound contents (TPC and TFC).

Our results show a strong and significant correlation between FRAP and DPPH (r = 0.986 ***), suggesting a consistent interplay between these two antioxidant capacities. Additionally, a high correlation among ABTS and TFC (r = 0.911 ***) was noted, reinforcing the implication of flavonoids in the scavenging activity captured by ABTS assay. Interestingly, DPPH also exhibited a robust positive correlation with TFC (r = 0.775 **), while FRAP showed a weaker but still significant correlation with TPC (r = 0.637 *), indicating phenolic compounds’ role in ferric reducing antioxidant potential.

However, some negative correlations stood out, such as the relationship between TFC and FRAP (r = −0.088) and between TPC and TFC (r = −0.045), suggesting a complex interaction in which flavonoid content may inversely affect certain antioxidant properties under specific conditions. These results are consistent across affected (CC1 and CC2) and unaffected (CS1 and CS2) samples, highlighting the influence of volcanic ash on metabolic and antioxidant dynamics in the potato crop.

Subgroup analyses revealed additional data. For example, the correlation between TPC and ABTS was particularly pronounced in unaffected samples (CS1: r = 0.963; CS2: r = 0.791) compared to affected samples (CC1: r = −0.384; CC2: r = −0.334). This suggests that volcanic ash may alter the relationship between phenolic content and antioxidant capacity. Furthermore, flavonoid content showed a stronger relationship with antioxidant capacity in ash-treated samples (e.g., TFC and ABTS, CC1: r = 0.659; CC2: r = 0.649), possibly reflecting an adaptive metabolic response to volcanic ash exposure.

### Inductive Plasma Coupled

The ICP analysis of potato samples from the Cotopaxi region revealed notable differences in elemental concentrations between tubers collected from unexposed soil (SC) and exposed soil (PCC). Potassium (K) was the most abundant element, with 17,215.34 mg/kg in control samples (CS1 and CS2) and 14,525.76 mg/kg in post-Cotopaxi eruption conditions (CC1 and CC2), followed by magnesium (Mg) at 661.28 mg/kg (SC) and 855.87 mg/kg (PCC). Calcium (Ca) and sodium (Na) showed moderate concentrations, while iron (Fe) increased from 104.47 mg/kg in soil control to 180.82 mg/kg in post-Cotopaxi eruption conditions. Other elements like boron (B) and copper (Cu) decreased post eruption, while aluminum (Al) showed a significant rise from 26.59 mg/kg in SC to 88.56 mg/kg in PCC. Metallic elements such as zinc, lithium, boron, manganese, barium, lead, nickel, chromium, and indium showed concentrations below 0.01 mg/kg.

**Figure 4:**
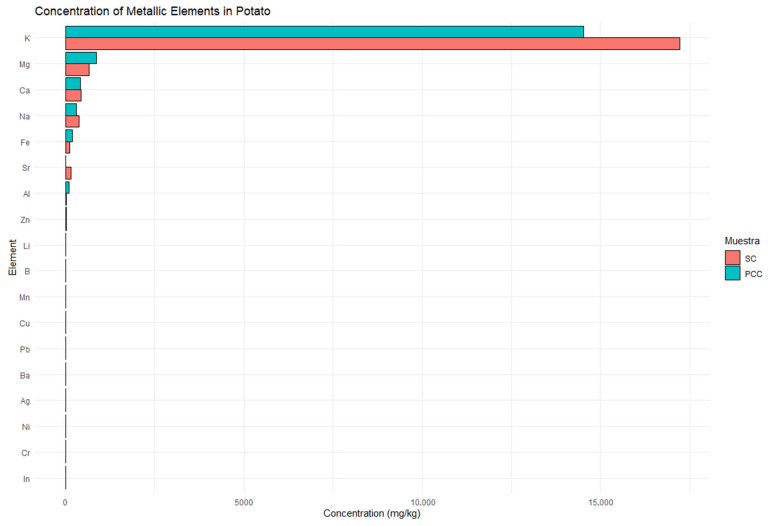
Concentration of metallic elements in potato samples. Legend: SC—tubers from the control soil; PCC—post-Cotopaxi eruption conditions. The *x*-axis represents element concentrations, while the *y*-axis lists the detected elements. Concentrations below 0.01 mg/kg of metallic elements were excluded from the visualization.

Overall, volcanic ash deposition influenced the elemental composition of potatoes, increasing Fe, Al, and Mn while reducing Cu, Ni, and B levels. These changes may impact the nutritional and metabolic properties of potato crops in the region (Figure 4).

**Figure 5:**
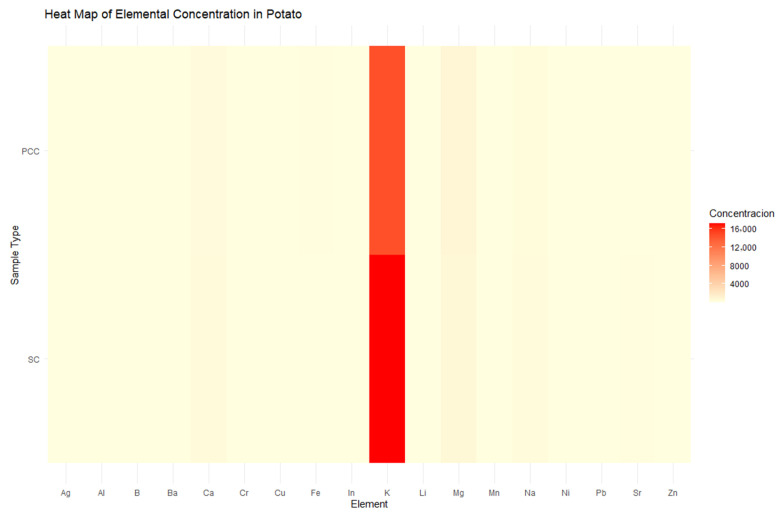
Heatmap of elemental concentration in potato samples (ICP). This heatmap visually represents the concentration levels of various metallic elements (mg/kg) in potato samples from the Cotopaxi region. The color gradient highlights the most and least concentrated elements, with potassium (K) standing out as the most abundant, followed by magnesium (Mg) and calcium (Ca). Elements with concentrations below 0.01 mg/kg were nearly undetectable and are not prominently displayed.

The ICP analysis of potato samples from the Cotopaxi region, visualized through a heatmap, confirmed that potassium (K) was the most abundant element, with concentrations of 17,215.34 mg/kg in soil-conditioned (SC) samples and 14,525.76 mg/kg in post-cultivation condition (PCC) samples, showing a noticeable decrease after cultivation. This trend is clearly reflected in the heatmap, where K appears as the most intense element. Magnesium (Mg) and calcium (Ca) followed in concentration, while elements like bismuth (Bi), cobalt (Co), and thallium (Tl) were nearly undetectable (<0.01 mg/kg). Transition metals such as iron (Fe) and zinc (Zn) showed moderate levels, with Fe increasing in PCC samples. Sodium (Na) and strontium (Sr) exhibited notable decreases, suggesting element mobilization during cultivation. The heatmap effectively illustrates the dominance of K and the minimal presence of other elements, reinforcing the influence of volcanic ash on elemental distribution and its potential implications for plant metabolism and nutritional quality (Figure 5).

## 4. Discussion

The present study demonstrates that potatoes cultivated in volcanic ash-influenced soils exhibit enhanced bioactive profiles compared to those grown in non-volcanic substrates.

The significantly higher total phenolic content in exposed samples (CC1 and CC2) suggests that the unique mineral composition and physicochemical properties of volcanic ash may act as abiotic stressors, triggering the biosynthesis of phenolic compounds. These compounds are well known for their antioxidant activity and implication in plant defense mechanisms, which can ultimately contribute to improved nutritional quality and stress resilience [20,21].

Furthermore, the marked variation in total flavonoid content observed, particularly in the CC2 samples, indicates potential metabolic adaptation to the heterogeneous nature of volcanic ash deposition. This variability might reflect the differential activation of stress response pathways that modulate flavonoid biosynthesis to counteract environmental fluctuations, such as changes in soil pH or the presence of heavy metals. Such adaptive metabolic responses could not only enhance the antioxidant capacity of the tubers but also elucidate the intricate relationship between plant secondary metabolism and soil chemistry [22].

Another crucial aspect explored in this study is the antioxidant content of potatoes, which reflects the plant’s defense mechanisms against external stressors. Antioxidants are critical for neutralizing reactive oxygen species, which can form under stressful conditions such as exposure to volcanic ash [9]. Interestingly, while volcanic ash represents a potential threat to food safety due to heavy metal contamination, its impact on plant stress responses may also have positive implications. The stress induced by ash exposure can potentially enhance the production of secondary metabolites, including antioxidants, especially phenolic compounds such as flavonoids, which contribute to the nutraceutical value of the tubers. These dual effects—both risks to food safety and potential benefits to nutritional quality—highlight the complexity of volcanic ash interactions with agricultural systems. The increase in antioxidant activity could be due to the upregulation of phenylalanine ammonia lyase (PAL) under ash-induced oxidative stress, promoting phenol synthesis [23]. However, this study is limited by the lack of transcriptomic data, detailed soil controls, and varietal specificity, which restricts the generalizability of the results.

The antioxidant assays (DPPH, ABTS, and FRAP) clearly demonstrate that volcanic ash-enriched soils significantly enhance the potatoes’ antioxidant activity. Specifically, the CC2 samples exhibited the highest radical scavenging activity in both the DPPH (4.80 ± 2.38 µmol Trolox g⁻^1^ DW) and ABTS (11.05 ± 2.57 µmol Trolox g⁻^1^ DW) assays, while CC1 showed superior reducing power in the FRAP assay (11.96 ± 4.57 µmol Fe^2^⁺ g⁻^1^ DW). These results imply that the unique volcanic ash mineral composition may stimulate the biosynthesis of bioactive compounds, thereby improving the overall antioxidant profile of the crop. Such results align with previous studies that have reported enhanced phytochemical accumulation in plants grown in volcanic substrates [24].

Furthermore, the marked differences in antioxidant activities between the samples underscore the complex interplay between soil properties and plant metabolic responses. The negative DPPH values observed in the unexposed samples (CS1), in contrast with the robust activity in the exposed samples (CC1 and CC2), indicate that potatoes grown without volcanic ash might lack sufficient defense mechanisms against oxidative stress. This disparity points to an adaptive response where the stress imposed by volcanic ash environments triggers the upregulation of antioxidant pathways, ultimately contributing to improved nutritional quality. Similar adaptive metabolic responses have been documented in the recent literature, emphasizing the potential of volcanic ash to serve as a natural enhancer of crop quality [23,25].

The correlation analysis reveals complex interactions among antioxidant capacity and bioactive compounds in potato cultivated in the Cotopaxi region. A remarkably positive correlation between FRAP and DPPH (r = 0.986, *p* < 0.001) indicates consistent interplay between reducing power and radical scavenging activity. In addition, the significant association between ABTS and TFC (r = 0.911, *p* < 0.001) reinforces the crucial role of flavonoids in scavenging free radicals, while the moderate correlation between DPPH and TFC (r = 0.775, *p* < 0.01) further supports the contribution of these compounds to antioxidant defense mechanisms. These findings align with recent studies emphasizing the pivotal role of secondary metabolites in enhancing plant antioxidant systems [22,23].

Interestingly, the analysis also revealed negative correlations between flavonoids and FRAP (r = −0.088) and between TPC and TFC (r = −0.045), suggesting a complex interaction that may be modulated by environmental conditions such as volcanic ash exposure. Subgroup analyses showed that, in samples cultivated without volcanic ash, the correlation between TPC and ABTS was particularly strong (CS1: r = 0.963; CS2: r = 0.791), whereas ash-treated samples exhibited inverse relationships (CC1: r = −0.384; CC2: r = −0.334). This divergence implies that volcanic ash could alter the synthesis or interaction of these bioactive compounds, potentially as an adaptive metabolic response to abiotic stress. Furthermore, the enhanced correlation between TFC and ABTS in ash-treated samples (CC1: r = 0.659; CC2: r = 0.649) underscores the possibility that volcanic ash may stimulate specific metabolic pathways involved in flavonoid biosynthesis [23,26].

Although the property of volcanic ash as a multi-nutrient mineral fertilizer has been demonstrated since 2010, in areas of Russia and Indonesia (two areas of the world that host very active volcanoes), its mechanisms remain monthly unknown [26,27].

The ICP analysis reveals that volcanic ash deposition in the Cotopaxi region significantly alters the elemental composition of potatoes. Notably, potassium (K) levels decrease in tubers collected from unexposed soil (SC) to 14,525.76 mg/kg in tubers collected from exposed soil (PCC), suggesting nutrient mobilization or altered uptake dynamics during cultivation. In contrast, the concentrations of magnesium (Mg) and iron (Fe) increase in PCC samples (from 661.28 mg/kg to 855.87 mg/kg and from 104.47 mg/kg to 180.82 mg/kg, respectively), indicating that volcanic ash may enhance the bioavailability of certain micronutrients by modifying soil pH and cation exchange capacity [25]. The significant rise in aluminum (Al) from 26.59 mg/kg to 88.56 mg/kg further underscores the transformative impact of volcanic ash on soil mineralogy, potentially influencing both plant stress responses and metabolic functions [25]. The levels of Fe (180.82 mg/kg) and Al (88.56 mg/kg) in exposed samples are below the FAO/WHO toxicity thresholds (Fe: 425 mg/kg; Al: 1000 mg/kg in tubers), suggesting safety for human consumption under current conditions.

These shifts in elemental profiles have critical implications for the nutritional and metabolic quality of potato crops. The enhanced levels of Fe and Mg in PCC samples may improve the crop’s nutritional value and support vital enzymatic processes associated with energy production and antioxidant defense [22]. However, the observed decreases of copper (Cu) and boron (B) (essential elements) raise concerns about potential deficiencies that could affect plant growth and metabolic balance. The heatmap visualization further corroborates these findings by illustrating the dominance of potassium alongside fluctuating trace element distributions, thereby highlighting the intricate relationship between volcanic ash inputs and nutrient uptake in potato cultivation. In our determinations, heavy metals appear to be in concentrations below 0.01 mg/kg, suggesting that heavy metals from the environment did not negatively influence the quality of potatoes in the area.

## 5. Conclusions

This study highlights the significant influence of volcanic ash on the nutritional and biological properties of potato tubers cultivated in the Cotopaxi region. Samples grown in volcanic ash-enriched soils demonstrated enhanced bioactive profiles. These findings suggest that the unique mineral composition of volcanic ash acts as an abiotic stressor, triggering adaptive metabolic responses that enhance the biosynthesis of secondary metabolites. The increased antioxidant activity observed in ash-treated samples, particularly in DPPH, ABTS, and FRAP assays, underscores the potential of volcanic ash to improve the nutraceutical qualities of crops. Elemental analysis further revealed that volcanic ash deposition alters the elemental composition of soils and tubers. While potassium (K) levels decreased, the increased concentrations of magnesium (Mg), iron (Fe), and aluminum (Al) indicate enhanced nutrient bioavailability and changes in soil chemistry. These shifts may contribute to improved nutritional quality but also raise concerns about potential heavy metal accumulation and food safety risks. The high variability in TFC observed in the CC2 samples reflects the heterogeneous nature of volcanic ash deposition and its potential to activate specific metabolic pathways in response to environmental stress.

Despite the potential benefits of volcanic ash exposure, such as increased antioxidant capacity, the findings raise critical questions regarding its long-term impact on crop safety and soil sustainability.

## Data Availability

The original contributions presented in the study are included in the article; further inquiries can be directed to the corresponding author due to privacy.
